# Supplementary material for: Unravelling reference bias in ancient DNA datasets
Source: Bioinformatics. 2024 Jul 3;40(7):btae436. doi: 10.1093/bioinformatics/btae436 (PMC11254355; doi:10.1093/bioinformatics/btae436)
Supplement: btae436_Supplementary_Data [file btae436_supplementary_data.zip › S1-S4_data_plots.zip!/S1-S4_data/read_origins_plots.zip!/read_origins_plots/bwamem_elephant_div2_1end_MQ1.pdf]

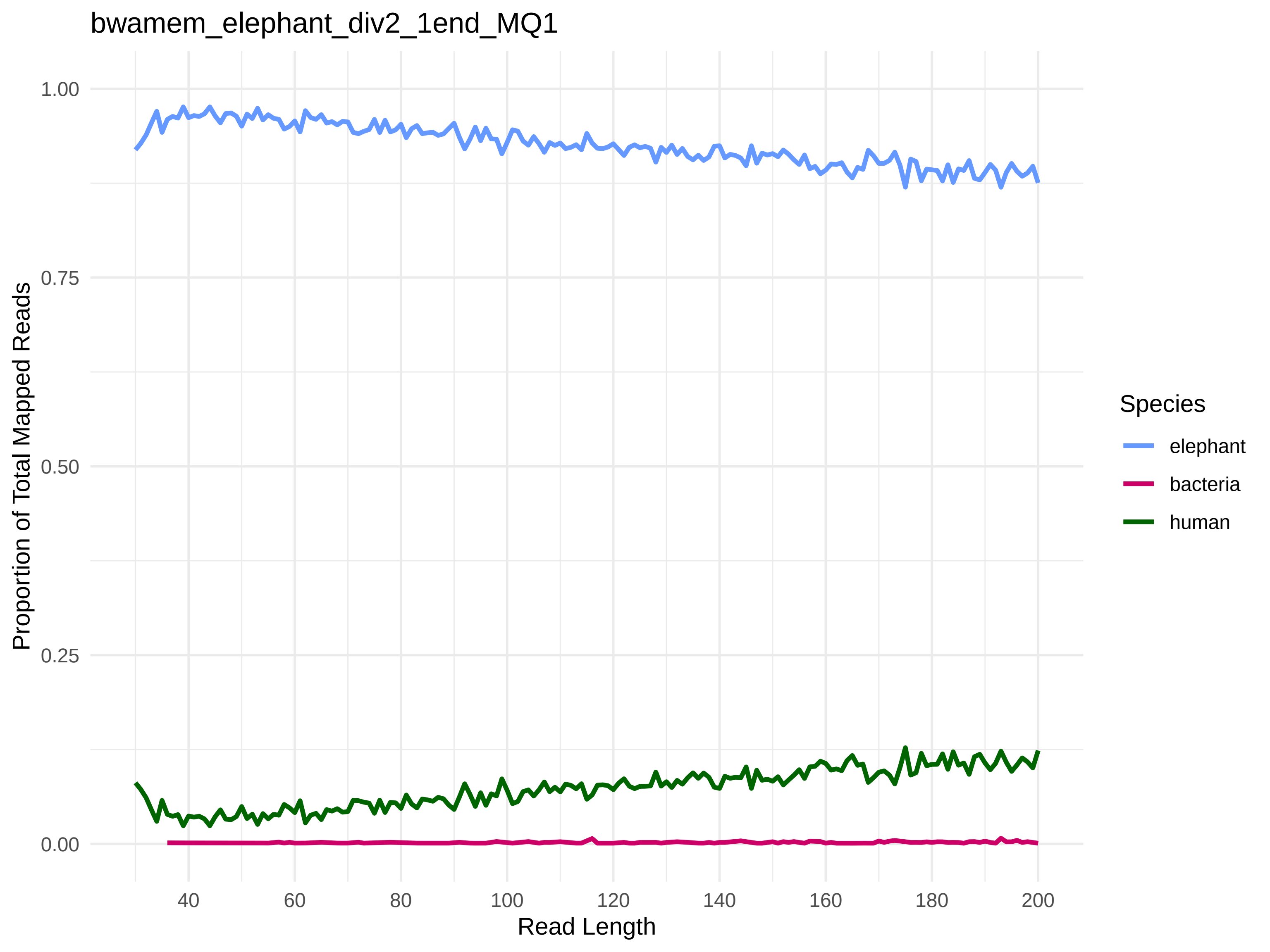
bwamem_elephant_div2_1end_MQ1
Proportion of Total Mapped Reads
1.00
0.75
0.50
0.25
0.00
Read Length
40
60
80
100
120
140
160
180
200
Species
elephant
bacteria
human
